# The Role of Hypoxia in Longevity

**DOI:** 10.14336/AD.2024.1630

**Published:** 2026-02-15

**Authors:** Ayesha Nisar, Sawar Khan, Yongzhang Pan, Li Hu, Pengyun Yang, Naheemat Modupeola Gold, Zhen Zhou, Shengjie Yuan, Meiting Zi, Sardar Azhar Mehmood, Yonghan He

**Affiliations:** ^1^State Key Laboratory of Genetic Evolution & Animal Models, Kunming Institute of Zoology, Chinese Academy of Sciences, Kunming, Yunnan 650201, China.; ^2^Kunming College of Life Science, University of Chinese Academy of Sciences, Kunming, Yunnan 650204, China.; ^3^Key Laboratory of Healthy Aging Research of Yunnan Province, Kunming Institute of Zoology, Chinese Academy of Sciences, Kunming, Yunnan 650201, China.; ^4^Department of Cell Biology, School of Life Sciences, Central South University, Changsha, Hunan 410083, China.; ^5^Institute of Molecular Biology and Biotechnology, The University of Lahore, Lahore 54000, Pakistan.; ^6^Department of Zoology, Hazara University, Mansehra 21300, Pakistan.

**Keywords:** aging, longevity, hypoxia, HIF-1, longevity-associated pathways, dietary restriction

## Abstract

Aging is marked by a progressive decrease in physiological function and reserve capacity, which results in increased susceptibility to diseases. Understanding the mechanisms of driving aging is crucial for extending health span and promoting human longevity. Hypoxia, marked by reduced oxygen availability, has emerged as a promising area of study within aging research. This review explores recent findings on the potential of oxygen restriction to promote healthy aging and extend lifespan. While the role of hypoxia-inducible factor 1 (HIF-1) in cellular responses to hypoxia is well-established, its impact on lifespan remains complex and context-dependent. Investigations in invertebrate models suggest a role for HIF-1 in longevity, while evidence in mammalian models is limited. Hypoxia extends the lifespan independent of dietary restriction (DR), a known intervention underlying longevity. However, both hypoxia and DR converge on common downstream effectors, such as forkhead box O (FOXO) and flavin-containing monooxygenase (FMOs) to modulate the lifespan. Further work is required to elucidate the molecular mechanisms underlying hypoxia-induced longevity and optimize clinical applications. Understanding the crosstalk between HIF-1 and other longevity-associated pathways is crucial for developing interventions to enhance lifespan and healthspan. Future studies may uncover novel therapeutic strategies to promote healthy aging and longevity in human populations.

## 1. Introduction

Aging is marked by the steady decline in physiological function and reserving capacity over time, leading to an increased vulnerability to diseases. It involves a complex interplay of diverse elements, encompassing environmental, genetic, and lifestyle factors. With aging, there is a progressive decline in various bodily systems, including cardiovascular, musculoskeletal, neurological, and immune systems [1, 2]. Aging represents a multifaceted phenomenon characterized by a blend of diverse and predictable changes [3], encompassing increased vulnerability to common ailments, such as cardiovascular disease, Parkinson's disease, diabetes, Alzheimer’s disease, osteoarthritis and osteoporosis [1, 4-7]. Despite some predictable facets, aging as a whole unfolds inconsistently or non-linearly [8].

Understanding the aging process is paramount for extending human longevity and improving the standard of living during old age. Unraveling the mechanisms underlying aging can assist in identifying potential interventions to delay or mitigate age-associated diseases and frailty [9]. This knowledge can result in the formation of strategies to foster healthy aging, enhance resilience to age-related stressors, and optimize preventive healthcare measures. Moreover, insights into the molecular, cellular, and physiological changes associated with aging may facilitate the discovery of novel therapeutic targets and interventions aimed at extending lifespan and promoting healthy aging [1, 10].

A significant surge in aging and longevity research was observed over the past decade, marked by unprecedented growth in the field [11, 12]. In the past few years, researchers have identified various key hallmarks of aging, which represent essential biological mechanisms underlying age-related decline and diseases [11, 13, 14]. The United Nations declared the current decade (2021-2030) as a period dedicated to promoting healthy aging, emphasizing the significance for global leaders and policymakers to focus on improving the well-being of older people, both now and in the future [15].

**Figure 1.**
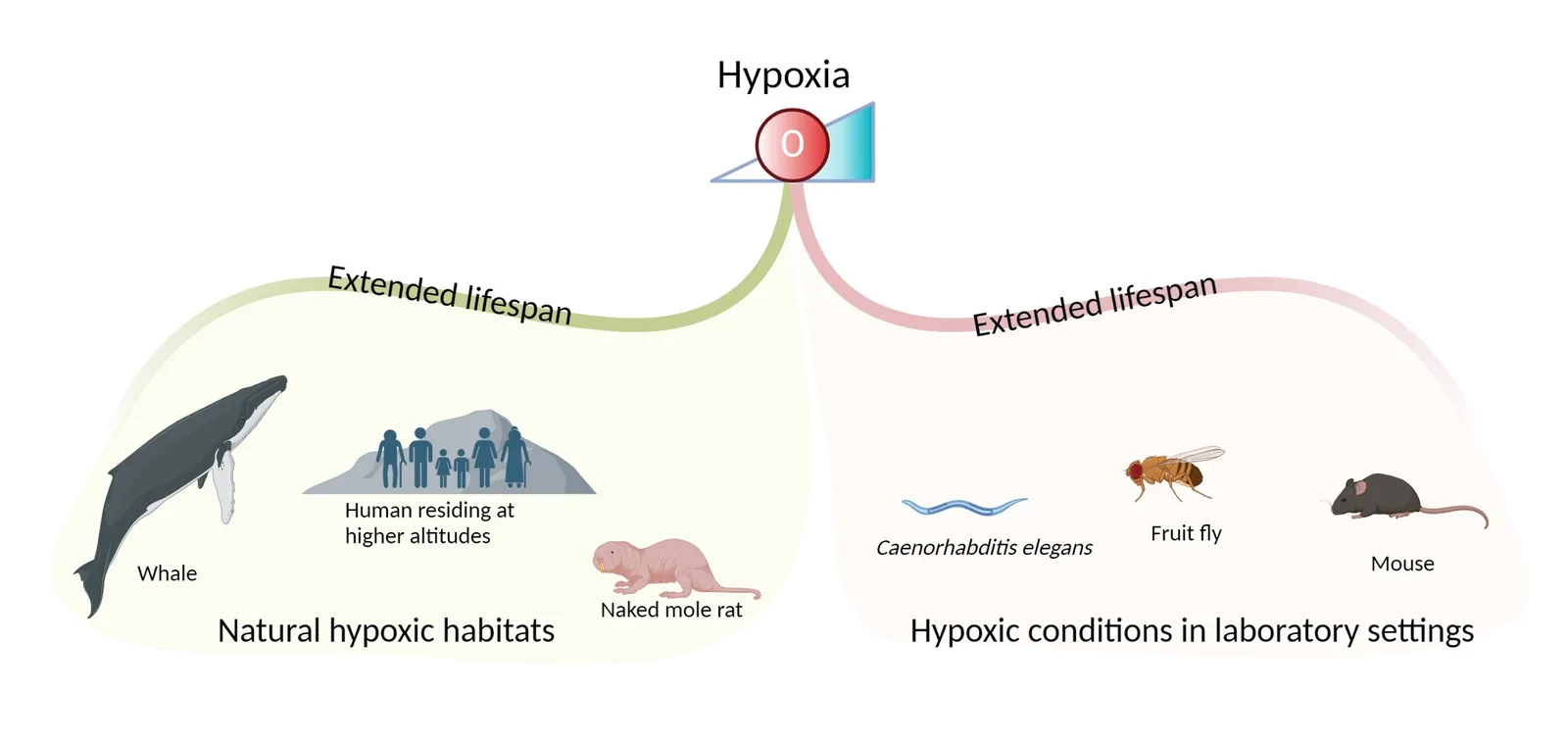
**Extended lifespan in different animal species under hypoxia in natural habitats and laboratory settings**. The potential link between hypoxia and lifespan extension in various species is illustrated. On the left, organisms that naturally thrive in hypoxic environments are highlighted, such as whales, humans residing at higher altitudes, and the naked mole rats. On the right, species exposed to hypoxic conditions in laboratory settings are shown, including *Caenorhabditis elegans*, fruit flies (*Drosophila melanogaster*), and laboratory mice. These examples underscore the potential role of hypoxia in promoting longevity.

As the global population ages at an unprecedented rate, the quest for innovative approaches to enhance healthspan and longevity intensifies. Longevity is influenced by various interconnected pathways including nutrient sensing pathways and other molecular players that impact aging processes and overall healthspan [16-22]. Hypoxia, characterized by diminished oxygen availability, has emerged as a compelling area of investigation within the realm of aging research [23]. The lifespans of model animals, such as *Caenorhabditis elegans*, have been demonstrated to increase in low-oxygen environments in laboratory settings [24, 25]. Furthermore, observations from the natural world reveal intriguing correlations between hypoxic habitats and extended lifespans in certain wild mammals (Fig. 1). For instance, species like the naked mole rat, which inhabits burrows with reduced oxygen concentrations, and marine mammals like whales, which must hold their breath during deep dives, exhibit remarkable longevity [26-28]. Additionally, elderly individuals residing at higher altitudes generally live longer than those at lower elevations [29]. These observations accentuate the potential role of hypoxia in modulating aging processes and suggest that adaptations to low-oxygen environments may confer longevity benefits. Previous studies have provided a broad overview of hypoxia and aging [30-32], as well as the effects of hypoxia in the context of heart diseases, including embryonic hypoxia [33, 34]. However, this review aims to offer a comprehensive and in-depth analysis specifically on the role of hypoxia in promoting longevity, detailing the underlying mechanisms and supported by illustrative graphics to enhance understanding.

Here, we will highlight recent findings on the potential benefits of oxygen restriction as an innovative approach to promoting healthy aging and increasing lifespan. We aim to comprehensively explore the current understanding of hypoxia-induced longevity, elucidating key findings, underlying mechanisms, and avenues for future research.

## 2. Cellular stress response and longevity

Stress adaptation is paramount for cell and organism survival as it enables them to navigate and respond effectively to a myriad of environmental challenges. Cells constantly encounter stressors, for example oxidative stress, heat shock, nutrient deprivation, and DNA damage, which can threaten their viability and function. In response, cells activate a sophisticated array of stress response pathways to mitigate damage, repair cellular components, and restore homeostasis. These adaptive responses involve the upregulation of molecular chaperones, activation of antioxidant defenses, induction of DNA repair mechanisms, and modulation of metabolic pathways. By mounting an appropriate stress response, cells can enhance their resilience, maintain functionality, and promote organismal survival in adversity.

Stress responses have been implicated in extending lifespan through various mechanisms that promote cellular health and resilience [35, 36]. One such mechanism is hormesis [37, 38], wherein exposure to mild stressors triggers adaptive responses that confer protection against subsequent, more severe stress. Additionally, stress responses can enhance cellular repair mechanisms, such as autophagy and proteostasis, thereby reducing the buildup of damaged biomolecules and organelles. Moreover, stress-induced activation of signaling pathways, for example the mTOR and insulin/IGF-1 pathways can modulate cellular metabolism and promote longevity. Furthermore, stress responses can stimulate the expression of longevity-associated genes and transcription factors, such as forkhead box O (FOXO) and sirtuin-1 (SIRT1). FOXO transcription factors are known to play a critical role in regulating cellular stress responses, promoting repair processes, and enhancing resistance to oxidative stress, thereby contributing to lifespan extension. SIRT1, a member of the sirtuin family, is involved in regulating metabolic processes, DNA repair, and inflammation, all of which are important for maintaining cellular function and longevity. These factors collectively contribute to enhanced cellular maintenance and increased lifespan.

Research involving *C. elegans* and other model organisms has offered valuable insights into the connection between cellular stress response and longevity [39-44]. Genetic and pharmacological manipulations in *C. elegans* have revealed conserved stress response pathways and longevity determinants that influence lifespan. For instance, mutations in insulin/IGF-1 signaling components, such as daf-2, extend the lifespan in *C. elegans* by activating stress response pathways like the FOXO transcription factor DAF-16 [45-47]. This pathway is conserved across species, including yeast, Drosophila, rodents, and humans [48]. In Drosophila, mutations in the insulin/IGF-1 pathway have similarly led to lifespan extension by activating FOXO transcription factors and enhancing stress resistance [49, 50]. Studies in yeast also show that reduced insulin/IGF-1 signaling contributes to lifespan extension through stress response mechanisms [51]. Moreover, research in rodents has demonstrated that dietary restriction (DR), a model for caloric restriction, activates similar pathways, resulting in enhanced stress resistance and extended lifespan [52]. Additionally, research in *C. elegans* and other model organisms has identified small molecules, including resveratrol and rapamycin, which mimic the effects of DR and promote longevity by enhancing stress resistance and cellular health [53]. The mechanisms underlying stress response and longevity identified in model organisms like *C. elegans* hold significant potential applicability to humans. Many of the molecular pathways and cellular processes involved in stress adaptation and lifespan extension are evolutionarily conserved across species. Therefore, interventions targeting these pathways, such as DR, exercise, and pharmacological modulators of stress response pathways, could potentially promote healthy aging and increase lifespan in humans. Moreover, insights gained from studying stress responses in model organisms can inform the formulation of novel therapeutic strategies for age-related diseases and disorders, ultimately improving human healthspan and quality of life.

Notably, one of the key pathways that exemplifies the intricate relationship between stress response and longevity is the hypoxic response pathway. This pathway, which will be discussed in detail in the next section, plays a crucial role in mediating the effects of low oxygen levels (hypoxia) on cellular and organismal longevity. The hypoxic response not only contributes to stress adaptation but also intersects with other longevity-promoting pathways, highlighting its significance in the broader context of aging and survival.

## 3. Hypoxic response pathway in longevity

Hypoxic exposure can be categorized as severe, mild, or chronic, with each type defined by specific oxygen concentration ranges, typically based on the partial pressure of oxygen (pO_2_) or oxygen saturation (SpO_2_) in tissues. Severe hypoxia is characterized by a pO_2_ of less than 30 mmHg or a SpO_2_ below 40% [54, 55]. Mild hypoxia occurs when the pO_2_ ranges from 30 to 60 mmHg, or the SpO_2_ is between 40-60% [56]. Chronic hypoxia typically refers to a sustained low oxygen environment, with pO_2_ values ranging from 60 to 80 mmHg or a SpO_2_ of around 60-90% [54, 57].

Severe hypoxia, marked by inadequate oxygen delivery to tissues, poses significant physiological challenges, as exemplified by high altitude sickness [58-61]. The deleterious effects of severe hypoxia include impaired cognitive function, tissue damage, and even death due to insufficient oxygenation of vital organs [62-65]. However, contrasting with this notion, mild hypoxia has been shown to have positive impacts on lifespan across various species. Studies have shown that exposure to mild hypoxia can induce a state of metabolic quiescence and promote cellular stress resistance, leading to increased lifespan [24, 25, 66]. Hypoxia triggers adaptive responses that enhance cellular survival by modulating energy metabolism, promoting autophagy, and reducing oxidative damage [64]. Moreover, changes in gene expression triggered by hypoxia can activate signaling pathways associated with longevity, such as the hypoxic response pathway mediated by the hypoxia-inducible factor (HIF)-1 protein [64, 67]. Understanding the significance of low oxygen conditions in extending lifespan provides insights into the complex interplay between environmental factors and cellular processes that regulate longevity.

### 3.1 Activation of the hypoxic response pathway and involvement of HIF-1 protein

Oxygen is indispensable for sustaining the viability and functionality of eukaryotic cells by serving as a pivotal component in mitochondrial energy production and as a necessary co-factor or substrate for numerous enzymes. When exposed to low oxygen levels, cells undergo a metabolic adaptation marked by a transition from predominantly mitochondrial metabolism to increased glycolysis, aiming to uphold adequate energy levels [68]. Subsequently, hypoxia triggers a signaling cascade primarily driven by the stabilization of hypoxia-inducible factors, notably the transcription factor HIF-1 [69, 70]. Activation of this pathway orchestrates the regulation of gene expression, governing various metabolic processes essential for cellular adaptation to hypoxic conditions.

**Figure 2.**
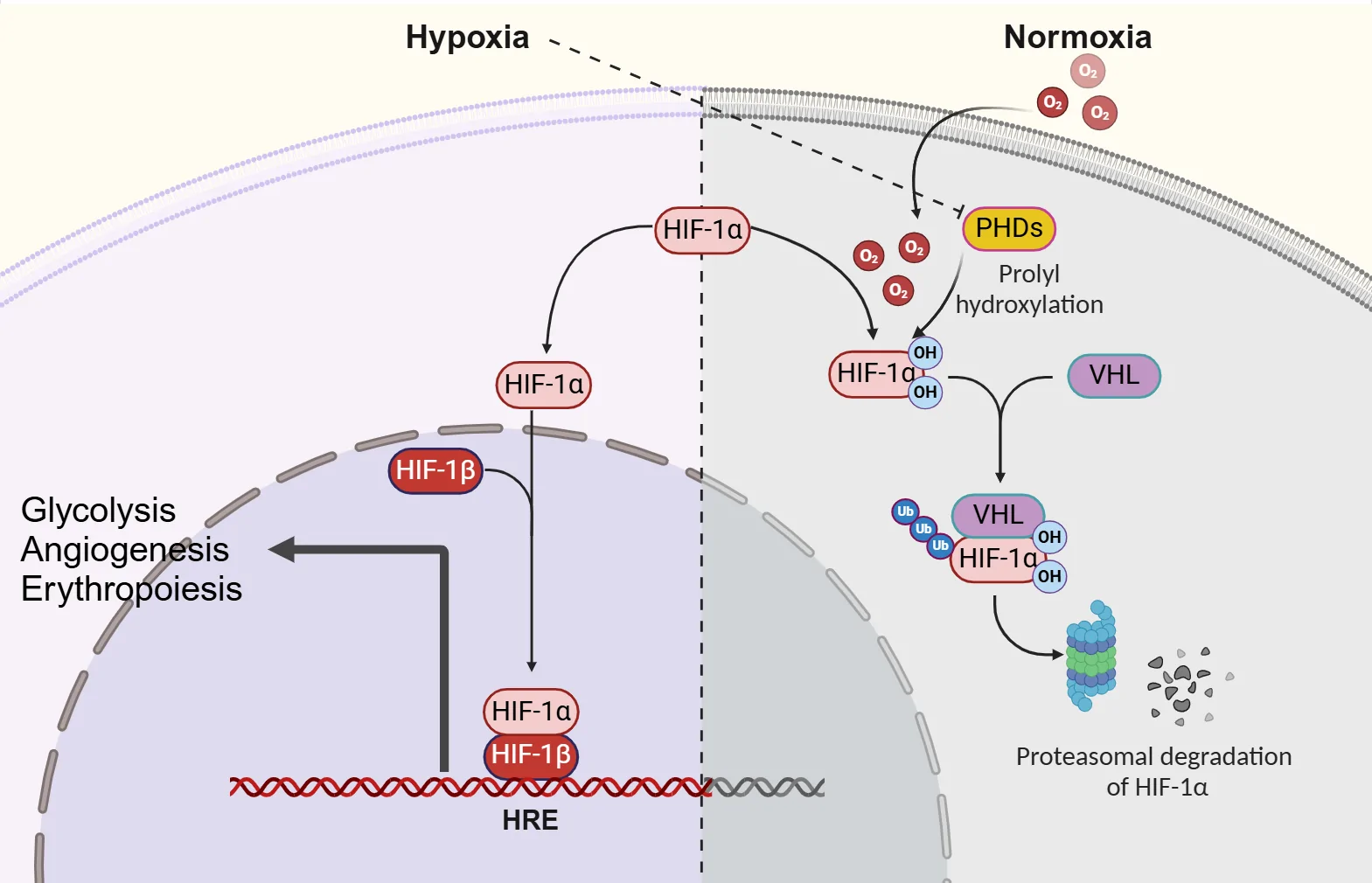
**The regulation of hypoxic response pathway**. The regulatory mechanisms of HIF-1α under hypoxic and normoxic conditions are illustrated. In hypoxia (left side), HIF-1α stabilizes and translocates to the nucleus, where it dimerizes with HIF-1β and binds to Hypoxia Response Elements (HRE) in the DNA, promoting the transcription of genes involved in various cellular processes such as glycolysis, angiogenesis, and erythropoiesis. In normoxia (right side), HIF-1α undergoes prolyl hydroxylation by prolyl hydroxylase domain enzymes (PHDs), facilitated by oxygen (O_2_). The hydroxylated HIF-1α then interacts with the von Hippel-Lindau protein (VHL), marking it for ubiquitination and proteasomal degradation. This degradation prevents HIF-1α from accumulating under normal oxygen conditions, maintaining cellular homeostasis.

The hypoxic response pathway is orchestrated by the HIF-1 protein (Fig. 2), which serves as a master regulator of cellular adaptation to low oxygen levels [71-73]. In normal oxygen conditions, HIF-1 undergoes hydroxylation by prolyl hydroxylase enzymes (PHDs), marking it for degradation through the ubiquitin-proteasome pathway, mediated by the von Hippel-Lindau tumor suppressor protein (VHL), which functions as the substrate recognition component of an E3 ubiquitin ligase complex [74-78]. However, in low-oxygen conditions, the activity of PHD is inhibited, resulting in the stabilization and accumulation of HIF-1 [76]. Stabilized HIF-1 moves to the nucleus, where it forms a heterodimer with HIF-1β and attaches to hypoxia-responsive elements (HREs) in the genome [79], initiating the transcription of target genes responsible for numerous cellular functions, including glycolysis, erythropoiesis and angiogenesis [68, 80, 81].

Activation of the hypoxic response pathway by HIF-1 enables cells to thrive in hypoxic environments and contributes to extending lifespan by interacting with various proteins associated with longevity in response to hypoxia. However, it is notable that despite the crucial role of HIF-1 in promoting cellular responses to low oxygen levels and extending lifespan, studies have revealed a decline in HIF-1 binding to HREs and expression of HRE-responsive genes with advancing age. For instance, research conducted on human cells, as well as liver, brain, lung, and kidney tissues in mice, along with the cerebral cortex of rats, has documented this age-related reduction in HIF-1 binding and HRE-responsive gene activation [82, 83]. To gain deeper insights into the mechanisms behind hypoxia-induced longevity, exploring the interplay between hypoxia and other established pathways and molecular players pertinent to aging is crucial. By elucidating how hypoxia intersects with these pathways and molecules, we can uncover synergistic or antagonistic interactions that may modulate the aging process and influence lifespan. This comparative analysis will not only enhance our comprehension of hypoxia-induced longevity but also provide valuable insights into the broader regulatory networks governing aging and longevity. Understanding the crosstalk between hypoxia and these pathways can shed light on potential targets for interventions aimed at promoting healthy aging and extending lifespan. In the subsequent section, we review the specific interactions and comparisons between the hypoxia pathway and other key pathways & molecular players implicated in longevity.

### 3.2 Distinct effects between hypoxia and dietary restriction (DR) on longevity

**Figure 3.**
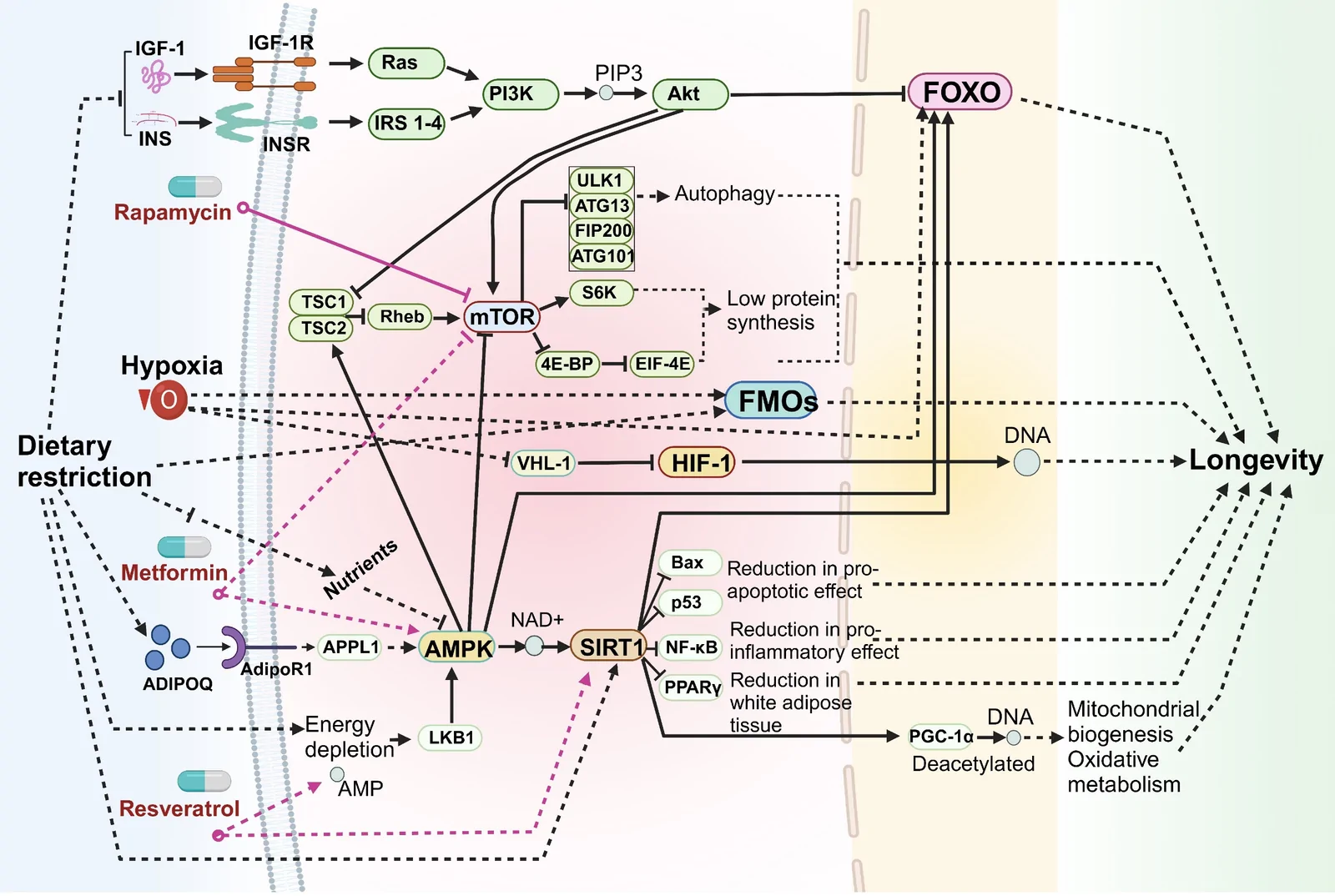
**Longevity regulating pathways and molecular players**. Regulation of longevity involves intricate interactions between genetic and environmental factors. Dietary restriction (DR), characterized by limited food intake, stands as the most well-established strategy for extending lifespan across the species. Four key pathways underline the effects of DR, including the IIS pathway, the AMPK pathway, the sirtuin pathway, and the mTOR pathway. These pathways promote longevity through activation of autophagy, stress defense mechanisms, and survival pathways, while simultaneously dampening proinflammatory mediators and cellular growth. Together, they enhance cellular fitness in response to DR. Hypoxia extends the lifespan independent of DR. However, both hypoxia and DR converge on common downstream effectors, such as FOXO and FMOs to modulate the lifespan. Additionally, pharmacologic agents like rapamycin, resveratrol, and metformin mimic the effects of DR, extending lifespan by targeting mTOR signaling, activating SIRT1 activity, and stimulating AMPK activity, respectively. These interconnected pathways collectively contribute to the modulation of lifespan and cellular homeostasis under varying conditions.

Dietary restriction (DR) is considered in animals as a well-researched and replicable approach for extending life expectancy [84]. Four pathways are recognized to mediate the DR effect, encompassing the insulin/IGF-1-like signaling (IIS) pathway, the AMP-activated protein kinase (AMPK) pathway, the sirtuin pathway, and the mammalian (mechanistic) target of rapamycin (mTOR) pathway. The collective response of these pathways to DR is believed to enhance fitness of the cells and, ultimately, extend lifespan (Fig. 3) by triggering autophagy, survival pathways, and stress defense mechanisms, while suppressing proinflammatory factors and cell proliferation [85].

Hypoxia exerts distinct effects on lifespan compared to other influential factors for longevity, such as DR. While DR involves reducing caloric intake without malnutrition, hypoxia represents a different environmental stressor that impacts cellular metabolism and stress response pathways. Extension of lifespan through DR differs from the stabilization of HIF-1, and the presence of HIF-1 is not essential for DR to enhance longevity [24]. DR activates nutrient-sensing pathways for example the IIS pathway and AMPK, leading to metabolic remodeling and increased stress resistance. In contrast, hypoxia triggers adaptive responses that optimize cellular function under oxygen-limiting conditions, promoting metabolic quiescence and cellular survival. Despite their differences, both hypoxia and DR converge on common downstream effectors, such as FOXO transcription factors [25, 41-44] and flavin-containing monooxygenases (FMOs) [86-88], to modulate lifespan, highlighting the intricate interplay between environmental cues and cellular processes in regulating longevity.

### 3.2.1 Crosstalk of HIF with IIS and AMPK pathways

The IIS pathway (the first nutrient-sensing pathway), initially discovered in *C. elegans* through mutations in *age-1* and *daf-2* genes, has been pivotal in understanding aging [45]. This pathway, conserved across various species including yeast, *C. elegans*, Drosophila, rodents, and humans, governs nutrient balance, growth, and longevity [48]. Activation of this pathway involves insulin-like peptide secretion in response to food cues, which then bind to insulin/IGF tyrosine kinase receptors [89]. Subsequently, the receptor transmits the signal to phosphatidylinositol 3-kinase (PI3K), catalyzing the conversion of phosphatidylinositol 4,5-biphosphate (PIP2) to phosphatidylinositol 3,4,5-triphosphate (PIP3) [90]. Elevated PIP3 levels activate protein kinases, prompting phosphorylation and cytoplasmic retention of FOXO transcription factors [46]. When insulin signaling diminishes, unphosphorylated FOXO translocates to the nucleus, facilitating the transcription of genes linked to longevity [91].

The hypoxic response pathway mediated by HIF-1 shares similarities with the IIS pathway in regulating longevity, albeit through distinct mechanisms [25]. While the IIS pathway responds to nutrient availability and regulates growth and metabolism, the hypoxic response pathway is activated in response to oxygen deprivation and modulates cellular adaptation to hypoxia. Both pathways converge on common downstream effectors that influence cellular metabolism and stress resistance, such as AMPK and FOXO. However, the specific context-dependent effects of each pathway on lifespan regulation differ, highlighting the complexity of the molecular mechanisms underlying longevity.

**Figure 4.**
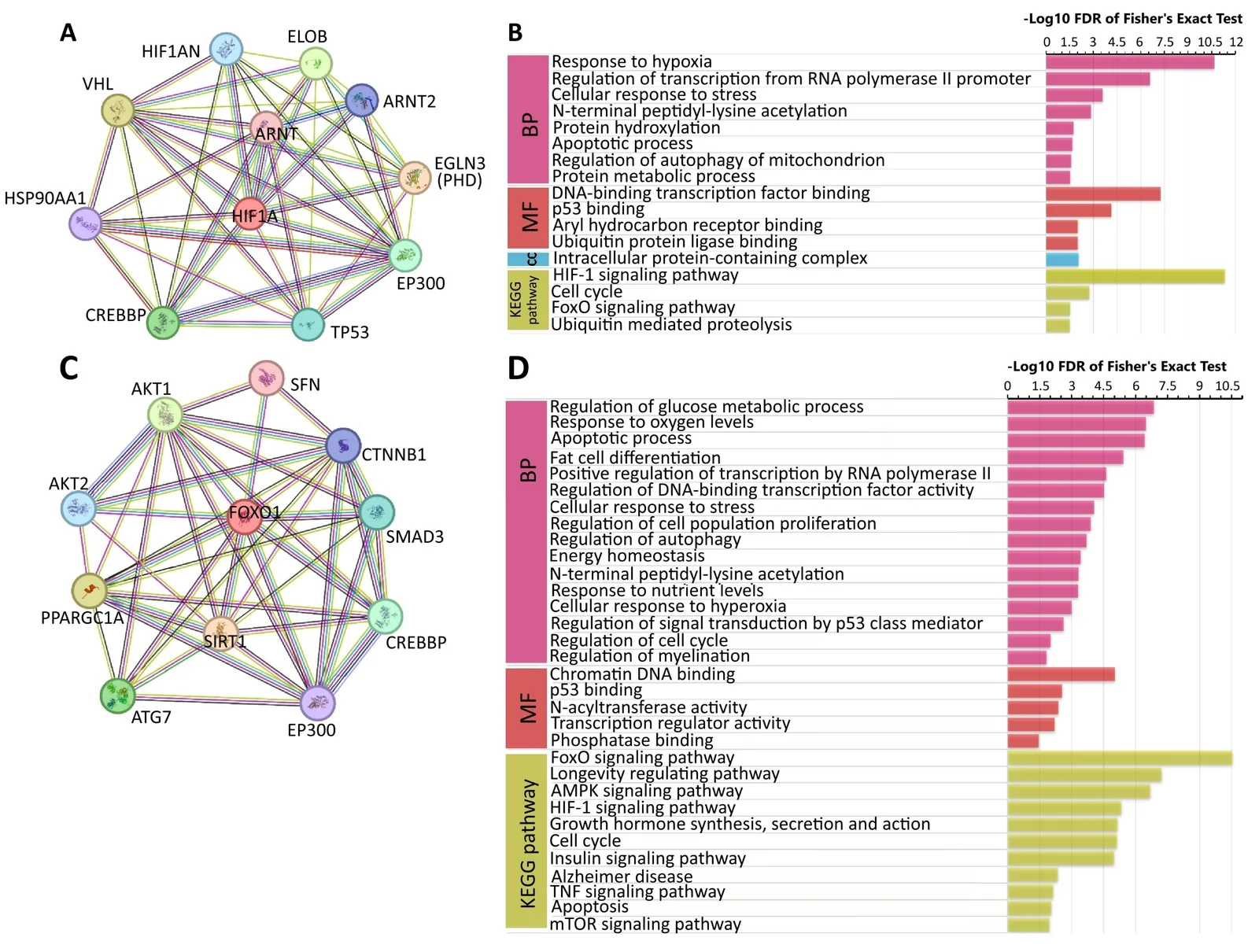
**Protein interaction network and GO terms enrichment analysis for HIF-1 and FOXO**. (**A**) Protein interaction network for HIF-1. (**B**) GO terms enrichment corresponding to HIF-1 network. (**C**) Protein interaction network for FOXO. (**D**) GO terms enrichment corresponding to FOXO network. The protein-protein interaction networks for HIF-1 and FOXO were constructed using the STRING database (https://string-db.org/). Nodes represent proteins, and edges represent interactions. Key proteins such as HIF1A, ARNT, and CREBBP for HIF-1; and FOXO1, AKT1, and SIRT1 for FOXO, are central in the networks. In GO terms enrichment analysis, the relevant terms and pathways for HIF-1 (like "Response to Hypoxia" and "HIF-1 signaling pathway") and FOXO (like "Cellular response to stress" and "FoxO signaling pathway") were significantly enriched, linking the networks to essential cellular responses under hypoxic conditions. BP: Biological Process; MF: Molecular Function; CC: Cellular Component.

The molecular interaction networks and associated GO terms were enriched for HIF-1 (Fig. 4A-B) and FOXO (Fig. 4C-D). The transcription factor FOXO has been implicated in promoting longevity by regulating various cellular functions such as metabolism, apoptosis, protein turnover and cell survival [92]. FOXO activation occurs in response to hypoxia, predominantly through a HIF-dependent pathway involving HIF-1α and, to a lesser extent, HIF-2α, in normal mouse and human fibroblasts, as well as various cancer cells. This FOXO activation under low oxygen conditions is linked to metabolic reprogramming, improved cell viability, and decreased production of reactive oxygen species (ROS) [93, 94]. Like HIF proteins, FOXO undergoes degradation by the proteasome after hydroxylation by PHD2, indicating an oxygen-dependent regulatory mechanism for FOXO [95].

There is consistent evidence of impaired activation of AMPK in response to hypoxia with aging [96]. Increasing AMPK levels have been demonstrated to prolong lifespan in both *C. elegans* and Drosophila [97, 98]. Additionally, metformin, an AMPK activator, has shown the ability to enhance healthspan and average lifespan in mice [99]. These findings suggest that AMPK serves as a key regulator of pro-longevity pathways, with many identified downstream interacting proteins. In *C. elegans*, fine-tuned interplay between HIF-1 and AMPK pathways governs lifespan [100]. AMPK functions as an "internal ROS quencher" by increasing thioredoxin levels, while HIF-1 facilitates ROS production through iron uptake, creating a delicate balance crucial for optimal stress resistance and longevity [100]. Furthermore, AMPK activates the longevity-promoting protein SIRT1 through both direct phosphorylation and increased NAD^+^ production via NAMPT enzyme stimulation [101, 102]. This SIRT1 activation further triggers downstream effectors like FOXO1 and FOXO3a, impacting stress resistance, metabolism, apoptosis and cell cycle [102]. Collectively, AMPK acts as a conductor, integrating signaling networks like HIF-1 and SIRT1 to promote health and delay aging [100-102]. However, declining AMPK activity disrupts this harmony, potentially accelerating the aging process.

### 3.2.2 Crosstalk of HIF with mTOR pathway

The mTOR suppression promotes lifespan across diverse organisms. This effect may be partially mediated by its regulatory influence on HIF-1α. Notably, the PI3K/Akt/mTOR pathway has been identified as a positive regulator of HIF-1α expression in both cancerous [103] and non-cancerous cells [104]. This interaction is facilitated by a specific motif within the N-terminus of HIF-1α, termed the TOS motif, which directly binds to the mTOR regulatory complex, Raptor [105]. Mutations disrupting this motif lead to impaired HIF-1α activity during hypoxia, highlighting its functional significance [105]. The relevance of this interaction extends to the realm of cancer biology. Aberrant activation of mTOR is frequently observed in tumorigenesis, leading to the buildup of HIF-1α and subsequent stimulation of cancer cell growth [106]. This association has been confirmed experimentally with the use of rapamycin, an inhibitor of mTOR, effectively inhibiting HIF-1α accumulation and its subsequent transcriptional activity in hypoxic PC-3 cancer cells [106]. Conversely, ectopic expression of wild-type mTOR further enhances HIF-1α activation in these cells, further solidifying the direct contribution of mTOR signaling to HIF-1α regulation.

The mTOR pathway and the hypoxia pathway are both significant regulators of cellular processes involved in lifespan extension. The mTOR pathway is important in coordinating the response of cells to nutrient availability and growth factor signaling, thereby influencing cell growth, proliferation, and metabolism. Suppressing mTOR activity has consistently correlated with increased lifespan in many species by promoting mechanisms such as autophagy [107-109]. Conversely, HIF-1 activation has been associated with lifespan extension potentially through its role in enhancing cellular stress resistance and promoting longevity pathways. Importantly, these two pathways exhibit significant crosstalk and mutual regulation, particularly in the context of cellular metabolism and stress responses. Hypoxia-induced inhibition of mTOR signaling can lead to reduced protein synthesis and increased autophagy, contributing to lifespan extension. Conversely, mTOR inhibition can activate HIF-1 and its downstream responses, such as glycolysis and angiogenesis, under conditions of hypoxia or nutrient deprivation. Thus, the complex interplay between the mTOR and hypoxia pathways represents a crucial nexus in the regulation of lifespan and cellular responses to environmental cues.

### 3.2.3 Crosstalk of HIF with cellular senescence regulation pathway

Cellular senescence, marked by an irreversible arrest of cell cycle, can be viewed as a two-step process: a temporary quiescence followed by a more stable geroconversion [110]. While CDKs (p16 and p21) are known regulators of the first step (G1-S arrest) [111], mTOR activity plays a crucial role in geroconversion [110]. Interestingly, Leontieva, et al. [112] demonstrated that hypoxic conditions, known to suppress mTOR, can prevent geroconversion even in the presence of p21 expression. This suggests that low oxygen can maintain a reversible quiescence, potentially due to decreased ROS production from mitochondria. This aligns with findings by Kondoh, et al. [113], who showed that increased glycolysis, resembling hypoxia, delays senescence in fibroblasts by reducing mitochondrial activity and ROS. Further supporting the link between cellular oxygenation and senescence, Casciaro, et al. [114] observed delayed senescence and improved functionality in amniotic fluid stem cells cultured at 1% O_2_, compared to atmospheric oxygen. A recent study [115] demonstrated that low-oxygen conditions lead to decreased expression of pro-inflammatory senescence-associated secretory phenotype through AMPK-mediated suppression of the mTOR-NF-κB signaling loop.

The crosstalk between the hypoxia pathway and cellular senescence is complex and context-dependent. Hypoxia can both promote and alleviate cellular senescence depending on the context [116]. Under chronic hypoxia, cells may enter a state of senescence as a protective mechanism against oxidative stress and DNA damage. This process involves the activation of HIF-1, which triggers the expression of genes related to cell cycle arrest and senescence, such as p21 and p16 [116, 117]. Additionally, HIF-1 can promote the expression of anti-apoptotic genes, allowing cells to survive in a senescent state. Conversely, acute or intermittent hypoxia has been shown to have anti-senescent effects [115, 118, 119] and may promote longevity. This phenomenon involves the activation of protective pathways that enhance stress resistance and promote cellular survival. Hypoxia-induced activation of HIFs can stimulate antioxidant defenses, DNA repair mechanisms, and autophagy, which collectively counteract cellular senescence and promote tissue homeostasis [33, 120]. Moreover, HIF-1 can inhibit the expression of pro-inflammatory factors linked to the senescence-associated secretory phenotype (SASP), thereby mitigating the harmful impact of senescent cells on the surrounding tissue microenvironment [121].

### 3.2.4 Crosstalk of HIF with sirtuin pathway

The sirtuin family, particularly SIR-2.1 in *C. elegans*, plays a critical role in regulating lifespan by promoting genomic stability and modulating metabolism according to nutrient availability [122-124]. SIR-2.1, a NAD^+^-dependent deacetylase, may influence HIF-1α activity through sirtuin-mediated deacetylation, affecting HIF-1α and other cofactors involved in HIF-1 signaling [125-127]. Notably, SIRT1, the mammalian homolog of SIR-2.1, suppresses HIF-1α activity under normoxic conditions by deacetylating lys674 of HIF-1α. However, this inhibition diminishes during hypoxia due to a decline in NAD^+^ levels, which results in reduced SIRT1 activity, increased acetylation of HIF-1α, and enhanced transcriptional activity of HIF-1α. This interaction is particularly relevant in aging, as the upregulation of SIRT1 in the brain has been associated with lifespan extension [128].

In experimental models involving mice exposed to hypoxia, reduced NAD^+^ levels and SIRT1 activity resulted in increased HIF-1α activity, contributing to a heightened rate of tumorigenesis in xenografts [127]. However, the replenishment of SIRT1 activity, either through gene alteration or resveratrol supplementation, has been shown to restore HIF-1α deacetylation in kidney cells exposed to hypoxia, subsequently reducing HIF-1-mediated gene expression [129]. This modulation suggests that the balance between SIRT1 and HIF-1α activity plays a key role in stress resistance and longevity under hypoxic conditions.

### 3.2.5 Molecular players in stress resistance and longevity and their potential interactions with the HIF-1 pathway

Several molecular players play critical roles in stress resistance and longevity, including SKN-1, CEP-1 and NF-κB. Understanding the potential interactions between these molecular players and the HIF-1 pathway provides insights into the integrated responses that govern resilience to stress and longevity in response to environmental cues such as hypoxia. SKN-1 is a transcription factor in *C. elegans* responsible for regulating gene expression related to detoxification pathways and antioxidant defense, thus enhancing stress resistance and promoting longevity [130-132]. CEP-1, the nematode homolog of p53, regulates apoptosis and stress responses, playing a dual role in promoting survival under stress and limiting lifespan extension [133-135].

The molecular players involved in stress resistance and longevity may interact with the HIF-1 pathway to coordinate cellular responses to environmental stressors. SKN-1, for instance, has been shown to intersect with HIF-1 signaling to regulate oxidative stress responses and metabolic adaptation to hypoxia [136, 137]. According to a recent investigation, the crosstalk between SKN-1 and HIF-1 is mediated by EGL-9 (a prolyl hydroxylase), where SKN-1 enhances the transcription of egl-9, consequently resulting in the inhibition of HIF-1 [138]. Additionally, CEP-1, the *C. elegans* homolog of p53, may crosstalk with HIF-1 to regulate cellular fate determinations during stress, balancing survival and apoptosis [133]. The interplay between p53 and HIF-1α is intricate and isn't simply linear; instead, a dynamic and context-dependent crosstalk governs cellular fate [139]. On one hand, p53, known as the "guardian of the genome," can suppress HIF-1α activity. Under severe hypoxia, p53 stabilization and increased transcriptional activity can lead to direct or indirect downregulation of HIF-1α, potentially promoting apoptosis [140-142]. Alternatively, HIF-1α can also influence p53 activity. Moderate oxygen stress results in the stabilization of HIF-1α and subsequent downregulation of p53 through various mechanisms, including transcriptional repression and competition for co-activators [143, 144].

Persistent mild inflammation significantly contributes to aging and the emergence of age-related ailments [145]. Many investigations have delineated HIF-1α's role in modulating NF-κB-driven cytokine release by macrophages and neutrophils, as well as in promoting cell viability in low-oxygen conditions [146, 147].

## 4. Experimental evidence in mammalian models

Recently, a study conducted by researchers at Massachusetts General Hospital explored the effects of oxygen restriction on longevity in mice [148]. The study revealed significant findings, demonstrating that oxygen restriction led to notable improvements in lifespan and delayed neurological decline in mice. Mice exposed to mild hypoxia exhibited an impressive 50% increase in lifespan compared to controlling animals maintained under normoxic conditions. Additionally, hypoxia-exposed mice showed delayed onset and progression of neurological decline, suggesting a protective effect against age-related neurodegenerative processes. Unlike calorie restriction, which is a well-studied method to increase lifespan, this is the first time that hypoxia has shown promise in a mammalian aging model. The mechanisms behind this lifespan extension by chronic continuous hypoxia are still unclear and more work is required to comprehend the exact mechanism and potential applicability to human aging.

Evidence from various mammalian disease models suggests that hypoxia holds therapeutic potential in mitigating pathological conditions. For instance, chronic continuous hypoxia has demonstrated considerable advantages in mouse models of Leigh syndrome, leading to improvements in survival, disease biomarkers, neuropathology, behavior, body temperature, and body weight [149]. Also, repeated acute intermittent hypoxia has demonstrated therapeutic efficacy in a rat model of spinal cord injury, indicating its potential for enhancing motor function recovery [150]. Moreover, hypoxia has been effective in correcting defects associated with mitochondrial diseases, such as reducing excessive molecular oxygen levels in *Ndufs4* knockout models and reinstating levels of Fe-S cluster in frataxin knockout models [151, 152]. In experimental models of multiple sclerosis, persistent hypoxia has been associated with enhanced vascular function, and apoptosis of infiltrating leukocytes [153]. A study in obese mice with type 2 diabetes found that a four-week intermittent hypoxia intervention significantly improved glucose homeostasis, insulin sensitivity, and GLUT4 translocation in skeletal muscle, suggesting hypoxia as a potential therapeutic approach for enhancing metabolic health [154]. Additionally, research on mice reported that intermittent exposure to hypoxic conditions contributed to significant weight loss and appetite suppression, indicating a possible role for hypoxia in regulating energy balance and reducing body weight [155]. These findings collectively suggest that hypoxia may exert pleiotropic effects or trigger convergent neuroprotective mechanisms, underscoring its potential as a therapeutic strategy across diverse disease contexts. However, extrapolating these findings to implications for longevity requires further exploration, considering the complex interplay of factors influencing aging processes.

In comparing oxygen restriction with other interventions known to extend lifespan, such as DR, several distinctions emerge. While DR involves reducing caloric intake without malnutrition, oxygen restriction represents a unique environmental intervention that modulates cellular metabolism and stress response pathways. Despite these differences, both interventions converge on common downstream effectors, such as the activation of stress resistance pathways and metabolic remodeling, contributing to lifespan extension. However, oxygen restriction offers the advantage of directly targeting oxygen-dependent processes and cellular metabolism, potentially providing additional benefits beyond those achieved with DR alone. Moreover, the observed delay in neurological decline in hypoxia-exposed mice suggests a distinct protective effect on brain health, underscoring the multifaceted benefits of oxygen restriction as a strategy for promoting healthy aging and longevity in mammalian models.

**Figure 5.**
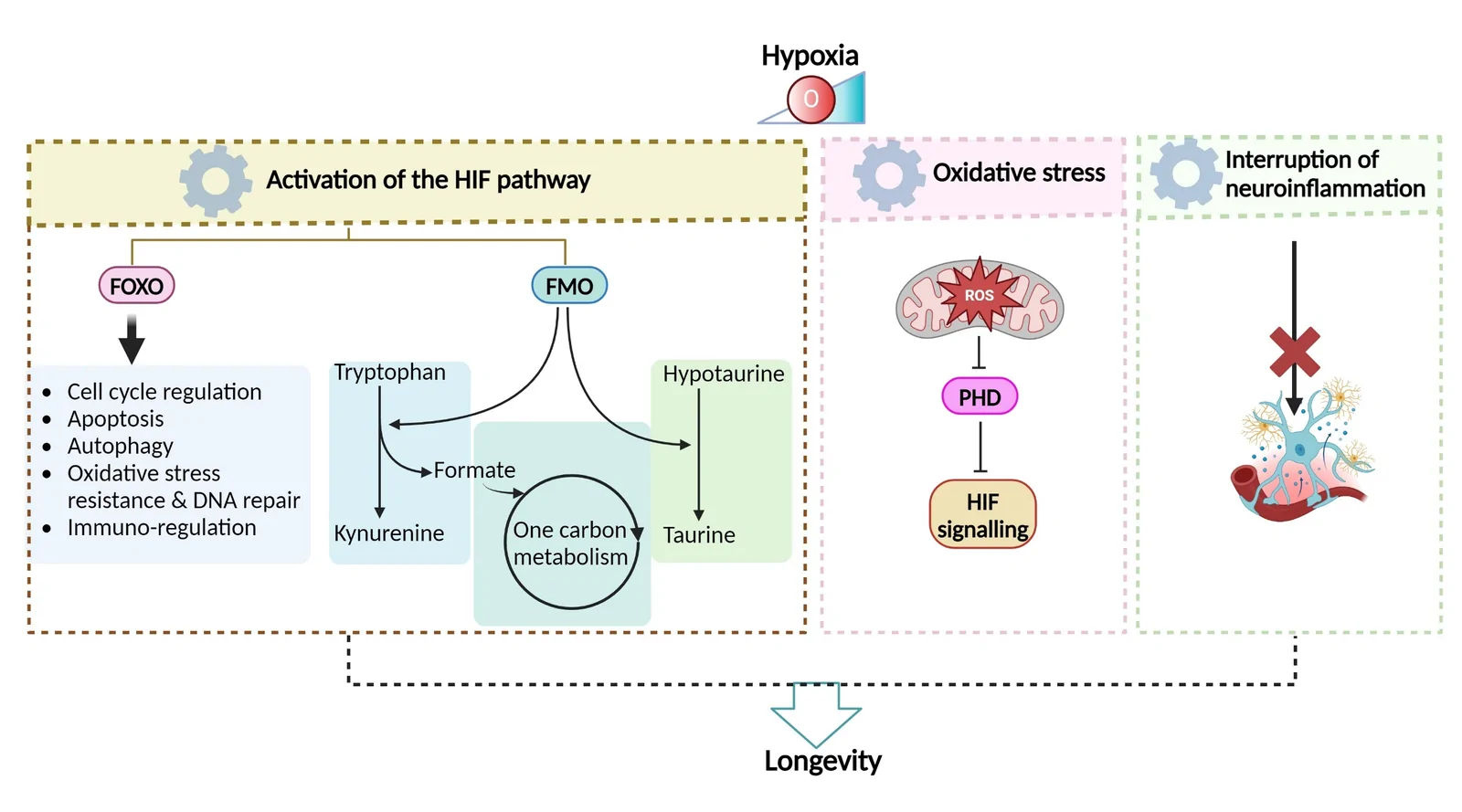
**Potential mechanisms underlying hypoxia-induced longevity**. On the left, the activation of the HIF pathway is shown, which converges the other known longevity-associated intervention the DR on common downstream effectors FMO and FOXO to modulate the lifespan. HIF interacts with FOXO to extend lifespan by regulating several crucial cellular processes, including cell cycle regulation, apoptosis, autophagy, oxidative stress resistance, DNA repair, and immunoregulation. FMO contributes to longevity via mechanisms involving the kynurenine pathway, taurine, and one-carbon metabolism. The middle panel highlights the role of mitochondrial ROS production, which inhibits prolyl hydroxylases (PHDs) and subsequently activates the HIF pathway. However, when ROS production exceeds the antioxidant system's capacity, oxidative stress damage occurs, leading to cellular dysfunction and pathological conditions. FOXO plays a crucial role in mitigating oxidative stress by promoting ROS elimination via superoxide dismutase (SOD) and catalase (CAT). On the right, the role of interrupting neuroinflammation in promoting longevity is emphasized. FOXO also plays a pivotal role in inhibiting neuroinflammation and promoting longevity by acting as a guardian of neuronal integrity in mammals. It achieves this by preventing age-related axonal degeneration, thereby safeguarding neuronal health and function. Additionally, FOXO enhances cellular resilience to oxidative stress by effectively reducing the accumulation of ROS, which may attenuate inflammatory responses in the nervous system, thus promoting overall cellular health and longevity.

## 5. Mechanisms underlying hypoxia-induced longevity

Some mechanisms have been proposed to underline hypoxia-induced longevity (Fig. 5), including activation of the HIF pathway, reduction of oxidative stress, and interruption of inflammation specifically neuroinflammation [148]. These proposed mechanisms collectively highlight the intricate interplay between hypoxia signaling pathways and cellular processes involved in aging and longevity regulation.

### 5.1 Activation of the HIF pathway

Activation of the HIF pathway represents a plausible mechanism underlying hypoxia-induced longevity. Hypoxia induces the activation and stabilization of HIF-1 transcription factor (section 3.1), which subsequently governs the gene expression linked to various processes, including metabolism, angiogenesis, and stress response. This activation facilitates cellular adaptation to low oxygen conditions and enhances stress resistance, thereby contributing to extended lifespan. Moreover, the activation of HIF-1 can intersect with other longevity-associated pathways, such as DR pathways (section 3.2). Both hypoxia and DR converge on common downstream effectors, such as FOXO, which is required to modulate longevity [46, 47]. However, it is essential to note that the specific context-dependent effects of each pathway on lifespan regulation differ, highlighting the complexity of the molecular mechanisms underlying longevity.

### 5.2 Reduction of oxidative stress

The reduction of oxidative stress emerges as a pivotal mechanism contributing to hypoxia-induced longevity, intertwined with the broader discourse on ROS and aging. The free radical theory of aging (FRTA) posits that oxidative damage inflicted by ROS constitutes a key driver of the aging process. Comparative studies and experimental interventions aimed at modulating ROS levels across various species have provided valuable insights into the intricate relationship between ROS and lifespan regulation. Surprisingly, the outcomes of these investigations reveal a complex interplay wherein ROS can exert divergent effects on lifespan depending on the experimental context [156].

### 5.2.1 Oxidative stress and redox biology

Redox biology plays a pivotal role in cellular homeostasis, influencing various physiological processes, including metabolism, signaling, and gene expression [157]. Central to redox biology is the balance between oxidants, such as ROS, and antioxidants, which act to neutralize ROS and prevent oxidative damage [158]. Dysregulation of redox balance leads to oxidative stress, a condition implicated in numerous pathological conditions, including cancer, neurodegenerative diseases, and cardiovascular disorders [159-162]. Understanding the intricate mechanisms of redox processes, ROS generation, and oxidative modifications is crucial for elucidating disease pathogenesis and developing therapeutic interventions.

### 5.2.2 Redox processes and ROS species

Redox processes involve the transfer of electrons between molecules, resulting in oxidation-reduction reactions. ROS encompass a diverse group of highly reactive molecules, including superoxide anion (O_2_^•-^), hydrogen peroxide (H_2_O_2_), hydroxyl radical (•OH), and singlet oxygen (^1^O_2_), among others [160, 161]. These ROS species are generated endogenously during cellular metabolism, primarily through mitochondrial respiration, enzymatic reactions, and peroxisomal activity. Exogenous sources, such as environmental pollutants and ionizing radiation, also contribute to ROS production. Importantly, ROS serve as signaling molecules in cellular pathways regulating proliferation, differentiation, and apoptosis [163, 164].

ROS can induce oxidative modifications in various biomolecules, including lipids, proteins, and nucleic acids. Lipid peroxidation, initiated by ROS attack on polyunsaturated fatty acids, leads to the formation of lipid hydroperoxides and reactive aldehydes, resulting in cellular membrane damage and dysfunction [165-167]. Protein oxidation involves the modification of amino acid residues, leading to protein misfolding, aggregation, and loss of function [168, 169]. Oxidative modifications to nucleic acids, particularly DNA, can cause mutations, DNA strand breaks, and genomic instability, contributing to carcinogenesis and aging [160, 170].

### 5.2.3 Hypoxia and redox processes

**Figure 6.**
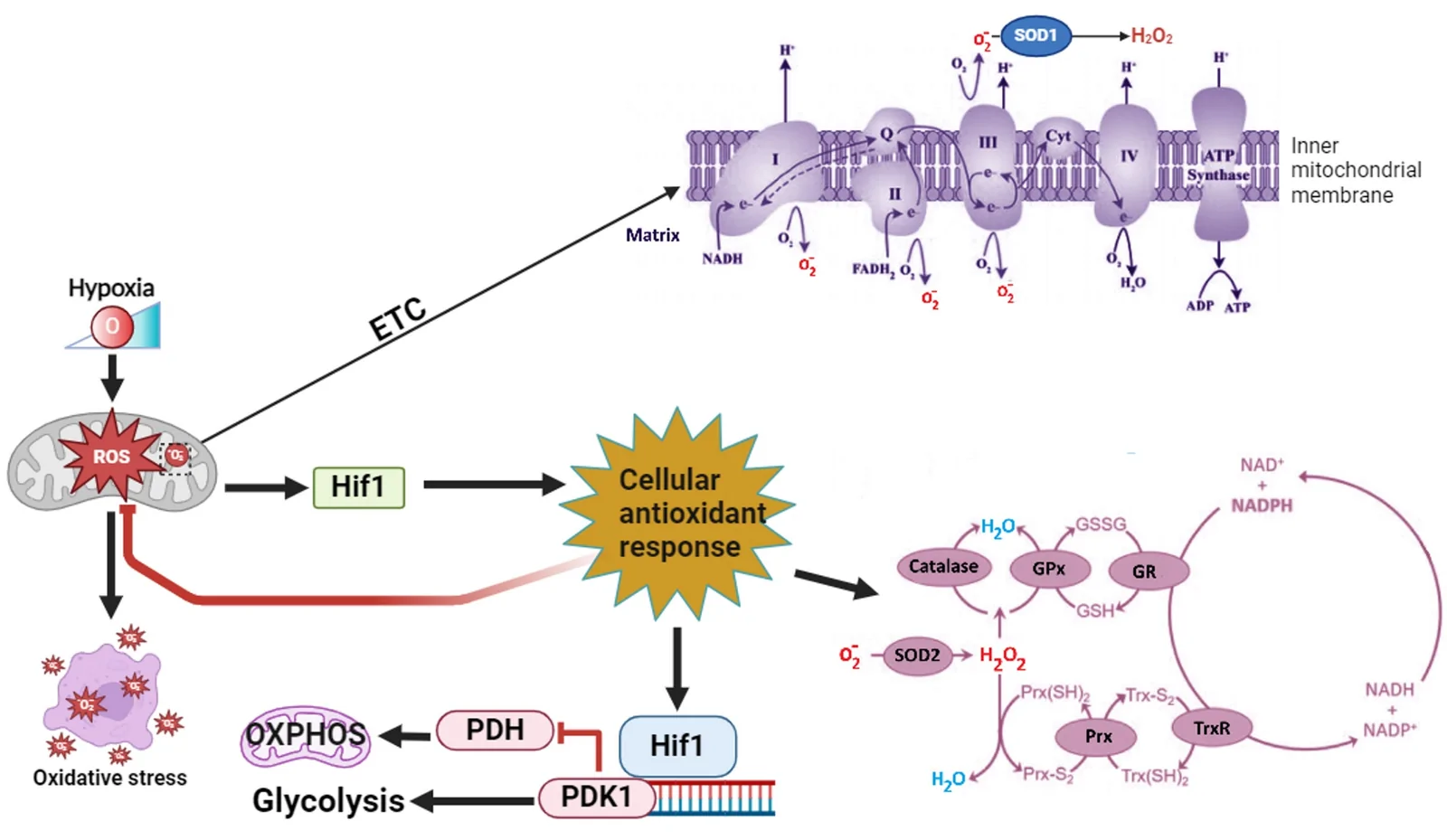
**Hypoxia and oxidative stress**. Relationship between hypoxia, reactive oxygen species (ROS), and oxidative stress, emphasizing the adaptive responses mediated by Hypoxia-Inducible Factor 1 (HIF1). Superoxide (O_2_•^-^) produced by the electron transport chain (ETC) is converted into hydrogen peroxide (H_2_O_2_) by superoxide dismutase (SOD), specifically MnSOD (SOD2) in the mitochondrial matrix and CuZnSOD (SOD1) in the intermembrane space. Catalase, glutathione peroxidase (GPx), and peroxiredoxin (Prx) further reduce H_2_O_2_ to water (H_2_O). GPx and Prx require glutathione (GSH) and thioredoxin (TrxSH) for their reduction processes, respectively. Glutathione disulfide (GSSG) is regenerated to GSH by glutathione reductase (GR), while oxidized thioredoxin (TrxS) is restored to TrxSH by thioredoxin reductase (TrxR). Both GR and TrxR utilize NADPH as a source of reducing equivalents. Hypoxia-induced ROS production stabilizes HIF-1α, which then activates the expression of pyruvate dehydrogenase kinase 1 (PDK1). PDK1 subsequently inhibits pyruvate dehydrogenase (PDH) function, blocking the entry of pyruvate into the mitochondria. This inhibition of PDH reduces oxidative phosphorylation (OXPHOS) and shifts cellular metabolism toward glycolysis. This process ultimately attenuates ROS production.

Hypoxia has profound effects on cellular redox homeostasis. It triggers adaptive responses essential for cell survival, primarily orchestrated by HIFs, which modulate gene expression in response to oxygen levels [171, 172]. Hypoxia contributes to longevity by reducing oxidative stress through complex mechanisms involving HIFs, mitochondrial adaptations, and antioxidant responses (Fig. 6). The interplay between hypoxia and ROS is intricate, involving feedback loops and cross-regulation that ultimately promote cellular adaptation and survival under low oxygen conditions. Understanding these processes is crucial for developing therapeutic strategies to leverage hypoxia and oxidative stress for health and longevity benefits.

### 5.2.4 Hypoxia-induced ROS production

Under hypoxic conditions, mitochondrial ROS production is enhanced due to electron leakage from the electron transport chain (ETC), particularly from complex I, II, and III [173-177]. Several studies validate the role of mitochondria in ROS generation during hypoxia. Cells depleted of mitochondria cannot produce ROS under hypoxic conditions, indicating the critical role of mitochondria in this process [178]. Specifically, transient bursts of superoxide induced by hypoxia rely on functional mitochondria, with mitochondrial complex III being a key player [179, 180]. Silencing complex III components mitigate hypoxia-induced ROS production, suggesting its role as a hypoxic sensor [179].

Transient mitochondrial ROS during hypoxia act as signaling molecules for HIF activation and cell proliferation [181]. Oxidative stress also acts as an additional stimulant for HIF activation [182]. PHDs, the primary regulators of HIF-1α stability, function as ROS sensors, with oxidative stress inhibiting PHD activity [183, 184]. The formation of oxidant-induced modifications in HIF-1α promotes its stabilization [185]. Consistent with these findings, mitochondrial ROS generated during hypoxia contribute to HIF upregulation [178, 186]. These findings underscore the interdependence of hypoxia and oxidative stress on HIF regulation, suggesting a network connecting these systems.

### 5.2.5 Cellular antioxidant response under hypoxia

Cells employ various mechanisms to suppress ROS and mitigate oxidative damage during hypoxia. HIFs regulate the expression of antioxidant enzymes such as superoxide dismutase (SOD), catalase, and glutathione peroxidase to scavenge ROS and maintain redox balance [187, 188]. Additionally, HIF-mediated metabolic adaptations, including a switch to glycolysis and metabolic reprogramming, reduce mitochondrial ROS production by limiting electron flow through the ETC. HIFs also enhance genomic stability by promoting the expression of DNA repair enzymes.

The interaction between hypoxia and oxidative stress involves HIFs and Nuclear factor erythroid 2-related factor 2 (NRF2). Hypoxic conditions trigger increased markers of oxidative stress, such as oxidized glutathione (GSH), resulting in heightened activity of antioxidant enzymes like glutathione peroxidases (GPXs) and SOD in various animal models [189]. This emphasizes the importance of understanding hypoxia stages and the mechanisms driving ROS production.

Prolonged exposure to hypoxia leads to excess ROS accumulation, posing a threat to cellular integrity. In response, HIF-1α upregulates pyruvate dehydrogenase kinase 1 (PDK1), promoting mitochondria-independent metabolism and enhancing electron transfer efficiency to curb ROS generation [190, 191]. HIF-1α also replaces complex IV subunits and inhibits complex I activity to further reduce mitochondrial ROS production during sustained hypoxia [190, 191]. Despite transient increases in ROS and antioxidants during hypoxia, NRF2 activation remains inconsistent across studies. For instance, NRF2 levels either remain unchanged or decrease in various cell types under hypoxic conditions, suggesting alternative mechanisms such as nuclear factor kappa B (NF-κB) and FOXO3 primarily mediate antioxidant responses [189, 192]. Additionally, HIF-1α activation inhibits NRF2 transcriptional activity in certain contexts [193, 194].

### 5.2.6 ROS and lifespan

Experimental manipulations that elevate ROS levels often lead to decreased lifespan, whereas interventions aimed at reducing ROS tend to extend lifespan. However, exceptions exist where increased ROS levels correlate with extended lifespan [156], suggesting a hormesis effect, where low-dose exposure to a stressor triggers adaptive responses [37, 38]. Hypoxia-induced reduction of oxidative stress may mitigate cellular damage and preserve physiological function, thus promoting longevity.

In various biological contexts, hypoxia modulates ROS signaling. For instance, in yeast and *C. elegans* models, hypoxia-induced ROS production has been linked with extended lifespan [195-197]. Similarly, in primary human lung fibroblasts, hypoxia-mediated ROS increase is associated with prolonged time to replicative senescence, indicative of enhanced cellular longevity [198]. These observations underscore the potential of moderate ROS increases under hypoxia to induce life-extending pathways.

### 5.3 Hypoxia and inflammation

Hypoxia plays a multifaceted role in regulating inflammation within the body. This role is characterized by its ability to either stimulate or suppress inflammatory responses, which can have significant implications for health, disease progression, and overall longevity [199]. The relationship between hypoxia and inflammation is well-documented, particularly through research on the hypoxia signaling pathway. For instance, in individuals experiencing mountain sickness, the increase in circulating proinflammatory cytokines, coupled with vascular leakage, can lead to pulmonary or cerebral edema [200-203]. Moreover, studies have shown that even healthy individuals exposed to high altitudes, such as 3400 meters or above, exhibit elevated serum levels of inflammation markers like interleukin-6 and C-reactive protein [204]. Extreme altitudes, such as those found on Mount Everest, have been associated with severe hypoxemia and increased alveolar-arterial oxygen differences, indicative of subclinical high-altitude pulmonary edema [202]. Animal studies further support these findings, showing that short-term exposure to low oxygen levels can result in vascular leakage, accumulation of inflammatory cells in various organs, and elevated cytokine levels [203, 205-207].

HIF-1α plays a key role in mediating the body's response to hypoxia and inflammation. Research has demonstrated that HIF-1α can inhibit the transcriptional activity of NF-κB under inflammatory conditions [208, 209]. NF-κB, a critical regulator of inflammation, is typically held inactive in the cytoplasm by inhibitory IκB proteins, which prevent it from binding to DNA [210-212]. Upon encountering inflammatory stimuli, the IκB kinase (IKK) complex phosphorylates IκB, leading to its degradation and allowing NF-κB to translocate to the nucleus, where it activates key inflammatory pathways [212-214]. Although the HIF pathway is primarily activated by hypoxia, it can also be upregulated by non-hypoxic factors such as bacterial lipopolysaccharide (LPS), tumor necrosis factor-α (TNF-α), ROS, and various cytokines, through interactions with the NF-κB pathway [215-217]. NF-κB subunits can directly bind to elements within the HIF-1α gene promoter region, enhancing its expression [218-221]. Moreover, both HIF and NF-κB pathways are regulated by similar mechanisms. For example, the IKK complex, which controls NF-κB activity, is targeted by PHD enzymes, making its activity sensitive to oxygen levels. Under normal oxygen conditions, PHD enzymes hydroxylate IKKβ, thereby suppressing NF-κB nuclear translocation and its transcriptional activity. Conversely, hypoxic conditions inhibit PHD activity, allowing the IKK complex to activate NF-κB by degrading IκB, leading to increased inflammatory gene expression [217, 222].

Inflammatory responses to hypoxia, particularly in the short term, serve as an adaptive mechanism that has evolved to enhance cell survival during conditions of infection, or injury [223, 224]. However, when hypoxia becomes chronic or systemic, it can lead to maladaptive inflammation, which in turn contributes to the development of various diseases. The interplay between hypoxia and inflammation is especially evident in severe conditions such as sepsis and trauma, where inflammation can exacerbate tissue hypoxia by increasing oxygen demand and reducing oxygen supply due to factors like edema and microthrombi [203, 225]. Conversely, hypoxia itself can modulate the immune response, producing either pro-inflammatory or anti-inflammatory effects depending on the context [226]. The immunomodulatory effects of hypoxia and hypoxia mimetics in animal models have yielded mixed results. Chronic hypoxia in mice has been associated with increased TNF-α levels following endotoxin challenge [227]. However, the hypoxia mimetic DMOG, which stabilizes HIFs, has shown anti-inflammatory effects in endotoxemic mice [228]. Furthermore, studies have indicated that acute hypoxia does not significantly alter the immune response but can increase the risk of morbidity and mortality from infections. Notably, preconditioning with prolonged hypoxia prior to infection has been found to mitigate these adverse effects and suppress the activation of inflammatory pathways in circulating leukocytes [229]. In critically ill patients, hypoxia is typically brief and is often quickly addressed through interventions like oxygen therapy or mechanical ventilation [230]. While the short-term effects of hypoxia on the immune system are not fully understood, research suggests that brief episodes of hypoxia may reduce systemic pro-inflammatory cytokine responses by enhancing purinergic signaling. This mechanism appears to be independent of HIFs and involves increased levels of adenosine, which then activate the adenosine 2B receptor, leading to a post-transcriptional increase in interleukin-10 (IL-10) production and a subsequent reduction in pro-inflammatory cytokine levels [226].

### 5.3.1 Neuroinflammation and aging

Chronic neuroinflammation is increasingly recognized as a significant contributor to the aging process and the development of neurodegenerative diseases. As the brain ages, microglia tend to adopt a more pro-inflammatory phenotype, characterized by a heightened release of cytokines and other inflammatory mediators even in the absence of overt pathology [231, 232]. This state of chronic low-grade inflammation, often termed "inflammaging" is linked to cognitive decline and increased susceptibility to neurodegenerative conditions such as Alzheimer's and Parkinson's diseases [233-236]. The suppression of neuroinflammation under mild hypoxia is particularly significant in the context of aging and longevity. The longevity benefits of hypoxia are not limited to the brain. Systemically, hypoxia can induce a range of adaptive responses that improve cellular stress resistance, enhance metabolic efficiency, and promote the maintenance of cellular homeostasis. These effects are thought to contribute to the lifespan-extending benefits observed in various organisms exposed to controlled hypoxic environments.

### 5.3.2 Interruption of neuroinflammation

Neuroinflammation, while essential in response to acute injury or infection, can become detrimental when chronic or unregulated, leading to neurodegenerative diseases. Interrupting this persistent inflammatory response is crucial for protecting neuronal health and preventing the progression of conditions like Alzheimer's and Parkinson's diseases. Hypoxia has been shown to interrupt neuroinflammatory processes implicated in age-related neurodegenerative diseases, potentially preserving cognitive function and extending lifespan. This notion is supported by evidence demonstrating its ability to alleviate severe neuroinflammation in mouse models of *Ndufs4* knockout, experimental autoimmune encephalomyelitis, and *Ercc1^Δ^*^/-^ conditions [153, 237]. Importantly, *Ercc1^Δ^*^/-^ models exhibit progressive microglial activation, a hallmark of neuroinflammation [238]. Brief and repeated exposures to mild or moderate hypoxia induce cellular and physiological adaptations known as intermittent hypoxia conditioning (IHC), which makes organisms more resistant to later hypoxic or ischemic challenges [239]. Studies have demonstrated that hypoxic preconditioning in mice, where animals were subjected to short periods of hypoxia before being exposed to a neuroinflammatory challenge, resulted in reduced microglial activation and lower levels of pro-inflammatory cytokines in the brain [240, 241]. This protective effect was mediated through the activation of HIF-1α and the subsequent induction of anti-inflammatory pathways. Such findings suggest that hypoxia, when carefully controlled, can be leveraged to modulate neuroinflammation in a beneficial manner. The observed attenuation of neuroinflammation holds significance, given its recognized role in amplifying neurodegeneration, thereby establishing a vicious cycle [242]. Yet, the precise workings by which hypoxia influences this cycle are still not entirely understood. It is uncertain if hypoxia functions by suppressing the inflammatory reaction to neuronal injury, providing neuronal resistance to the stress of DNA damage and inflammation, or through a blend of both processes [148]. Nonetheless, these findings imply that hypoxia-induced interruption of neuroinflammation may represent a promising avenue for preserving cognitive function and extending lifespan in the context of age-related neurodegenerative conditions.

### 5.3.3 Mechanisms to suppress neuroinflammation

Interestingly, mild hypoxia, when carefully controlled, can suppress neuroinflammation by activating protective pathways that enhance mitochondrial function, reduce oxidative stress, and promote autophagy. These processes collectively contribute to neuroprotection, potentially extending lifespan and improving cognitive function, especially in the context of aging. This suppression is largely mediated through the activation of anti-inflammatory pathways that counterbalance the initial pro-inflammatory response. For example, under conditions of mild hypoxia, HIF can induce the expression of anti-inflammatory mediators such as IL-10 and TGF-β, which serve to dampen the inflammatory response and promote tissue repair and homeostasis [226, 243]. HIF-1 increases IκBα expression, which inhibits NF-κB by preventing its nuclear translocation and reducing its inflammatory activity. HIF-1 and NF-κB compete for the coactivator p300, diminishing NF-κB's gene activation. This competition and the enhanced inhibition by IκBα help HIF-1 exert an anti-inflammatory effect [244].

Hypoxia also contributes to mitochondrial protection, a key factor in cellular health and longevity. Under hypoxic conditions, cells adapt by enhancing mitochondrial efficiency and reducing the production of ROS, which are major contributors to oxidative stress and neuronal damage. By lowering oxidative stress, hypoxia mitigates one of the primary drivers of aging-related cellular damage. Additionally, hypoxia has been shown to enhance autophagy, a process crucial for the removal of damaged organelles and proteins. FOXO, the downstream effector in the HIF pathway, is essential in potentially mitigating neuroinflammation and extending longevity by acting as a key protector of neuronal integrity in mammals [245]. It accomplishes this by inhibiting age-related axonal degeneration, which helps preserve neuronal health and functionality over time [246]. In addition, FOXO boosts cellular resilience to oxidative stress by efficiently reducing the accumulation of ROS. This action not only helps to lower inflammatory responses within the nervous system but also supports overall cellular health, contributing to prolonged longevity and potentially mitigating the effects of neurodegenerative diseases [246]. Through these mechanisms, FOXO plays a critical role in maintaining neuronal integrity and promoting a healthier aging process. Autophagy plays a vital role in maintaining cellular homeostasis, particularly in neurons where the accumulation of damaged proteins can lead to neurodegenerative diseases. Through enhanced autophagy [247], hypoxia supports the clearance of cellular debris and the recycling of cellular components, which may contribute to both the suppression of neuroinflammation and the promotion of longevity.

## 6. Challenges in understanding the precise molecular basis underlying hypoxia-induced longevity

Despite significant progress in elucidating the mechanisms underlying hypoxia-induced longevity, several challenges remain in understanding the precise molecular basis of this phenomenon. One challenge stems from the complexity of hypoxia signaling pathways and their crosstalk with other cellular pathways underlying aging and longevity regulation [64, 117, 248, 249]. Disentangling the specific contributions of individual components within the hypoxia response network presents a formidable task [250, 251]. Moreover, the context-dependent effects of hypoxia on different cell types and tissues further complicate efforts to delineate its molecular basis [252, 253]. Additionally, variations in experimental conditions, such as the degree and duration of hypoxic exposure [23], pose challenges in interpreting study outcomes and extrapolating findings to physiological relevance. Addressing these challenges will require interdisciplinary approaches integrating molecular biology, systems biology, and computational modeling to gain a comprehensive understanding of hypoxia-induced longevity.

## 7. Populations study at high altitudes and practical hypoxia regimens

Exploring the potential benefits and applicability of hypoxia-induced longevity in human populations is of great interest for developing strategies to promote healthy aging and improve overall healthspan [254]. While studies in model organisms have demonstrated promising effects of hypoxia on lifespan extension and healthspan improvement, translating these findings to human populations requires careful consideration of various factors, including safety, efficacy, and practicality. Hypoxia-based interventions, such as intermittent hypoxia training or hypoxia-mimicking drugs, hold the potential to enhance cellular resilience, reduce chronic disease risk, and extend lifespan in humans. However, rigorous clinical trials are needed to evaluate the safety and effectiveness of these interventions in diverse human populations, considering factors such as age, sex, genetics, and baseline health status.

Studying populations living at high altitudes provides valuable insights into the effects of chronic hypoxia on human physiology and longevity. High-altitude populations, such as those residing in the Andes Mountains or Tibetan Plateau, have adapted to hypoxic environments over generations, exhibiting unique physiological traits that confer resilience to low oxygen conditions [23, 29, 255-258]. By investigating the genetic, epigenetic, and metabolic adaptations of high-altitude populations, investigations can uncover novel mechanisms underlying hypoxia-induced longevity and identify potential targets for intervention. Furthermore, exploring practical hypoxia regimens, such as intermittent hypoxia training [259-261] or hypoxia-inducing pharmaceuticals [262-265] (Table 1), offers opportunities to harness the benefits of hypoxia-induced longevity in a controlled and reproducible manner. Investigating the impact of these hypoxia regimens on human health and lifespan in diverse populations can inform the development of personalized interventions tailored to individual needs and preferences, ultimately advancing efforts to promote healthy aging and enhance longevity in human populations.

**Table 1:** Hypoxia-inducing agents.

Type	Category	Description	Compound	References
**PHD inhibitors**	Iron competitors	Compete for iron ions to bind the active site of PHD	CoCl_2_	[265, 279-285]
	Iron chelators	Diminish the pool of free iron ions accessible for catalyzing the hydroxylation of HIF-1	DFO	[282, 284, 285]
			Deferiprone (CP20)	[282]
			Deferasirox (Exjade)	[282]
			Hydralazine	[265, 286]
	2-OG analogs	Inhibit the activity of PHDs by competing with PHD substrates, such as 2-OG	DMOG	[281-283, 285]
			IOX2	[282]
			FG4592 (Roxadustat)	[262, 264, 282]
			GSK1278863 (Daprodustat)	[264]
			BAY 85-3934 (Molidustat)	[264, 287]
			GSK360A	[262]
	Oxygen depleter/Enzymatic hypoxia	This system operates with the enzymes GOX and CAT. GOX facilitates oxygen depletion by oxidizing glucose into gluconolactone and H_2_O_2_ (ROS). Meanwhile, CAT metabolizes H_2_O_2_ into water and oxygen (half a molecule) to mitigate cytotoxic effects resulting from ROS accumulation. Overall, this system consumes oxygen and induces hypoxia.	GOX/CAT system	[280, 288]
Non-PHD inhibitors	-	Anesthetic	ISO	[289]
		Not specified	DNP	[289]
		microRNA	miR-335	[290]
		Antioxidant	NAC	[291]
		Proteasome inhibitor	MG-132; Epoxomicin	[292]
		Proteasome inhibitor	BSc2118	[293]
		Antiviral agent	Tilorone	[294]

Abbreviations: CAT, Catalase; CoCl_2_, Cobalt chloride; DFO, Desferrioxamine; DMOG, Dimethyloxalylglycine; DNP, 2,4-dinitrophenol; GOX, Glucose oxidase; HIF-1, Hypoxia-inducible factor-1; ISO, Isoflurane; PHDs, Prolyl hydroxylases; 2-OG, 2-oxoglutarate

In addition to studying high-altitude populations, examining species that have evolved to thrive in low-oxygen environments, such as the naked mole rat and marine mammals like whales, offers valuable insight into the physiological adaptations that support longevity in hypoxic conditions. The naked mole rat, a subterranean rodent living in low-oxygen, high-carbon dioxide environments, has developed remarkable resistance to oxidative stress [266]. Despite these harsh conditions, it exhibits an extended lifespan and extraordinary cellular resilience [267]. Research has shown that the naked mole rat's ability to prevent oxidative damage is key to its longevity and helps it maintain homeostasis despite low oxygen availability [268-272]. Interestingly, stabilized HIF-1α has been observed in NMRs under normoxic conditions [267], which is surprising and contrasts with other organisms, where HIF-1α is typically degraded under normal oxygen levels. Similarly, marine mammals, such as whales and seals, experience intermittent hypoxia during deep dives and have evolved unique mechanisms to mitigate hypoxic effects [273-276]. These adaptations include enhanced oxygen storage, efficient metabolic shifts, and increased antioxidant defenses, all contributing to their longevity and overall healthspan. For example, the bowhead whale, which faces repeated episodes of hypoxia during dives, exhibits reduced oxidative damage and enhanced oxidative stress resistance, enabling it to live longer despite these challenges [277, 278]. The adaptations seen in both the naked mole rat and marine mammals underscore the potential for hypoxia-induced longevity. These species provide crucial comparative models for understanding how hypoxia influences lifespan and healthspan.

### 7.1 Investigating combined effects and interactions of oxygen restriction with other interventions

Understanding the potential interactions between hypoxia-induced longevity and known anti-aging interventions is essential for optimizing strategies to promote healthy aging and extend lifespan. Exploring the combined effects of oxygen restriction with DR or chemical compounds offers promising avenues for enhancing longevity and healthspan. Both oxygen restriction and DR have been demonstrated to trigger stress response pathways and promote metabolic remodeling, leading to improved cellular function and extended lifespan via common downstream effectors such as FOXO and FMOs [25, 41-44, 86-88]. By combining these interventions, their beneficial effects on aging and longevity can potentially be amplified. Moreover, exploring the effects of hypoxia-mimicking chemical compounds, such as HIF stabilizers [262-265, 295] or mitochondrial modulators [296], in conjunction with oxygen restriction or DR, provides opportunities to target multiple pathways involved in aging and age-related diseases.

Investigating the combined effects of oxygen restriction with DR or chemical compounds involves assessing changes in gene expression, metabolic activity, and physiological function to elucidate the underlying mechanisms governing their synergistic interactions. By elucidating the potential synergies or conflicts between hypoxia and known anti-aging interventions, future work can identify optimal strategies for promoting healthy aging and enhancing longevity in diverse contexts.

### 7.2 Potential benefits and risks associated with hypoxia-induced longevity

While hypoxia-induced longevity holds promise as a potential intervention for promoting healthy aging, it is essential to consider both its potential benefits and associated risks. The activation of the HIF pathway and reduction of oxidative stress under hypoxic conditions may confer protective effects against age-related diseases and extend lifespan. Furthermore, interruption of neuroinflammatory processes by hypoxia could preserve cognitive function and improve overall healthspan. However, prolonged or severe hypoxic exposure may pose risks, including tissue damage, impaired physiological function, and exacerbation of certain pathological conditions [32, 297, 298]. Moreover, the optimal duration and intensity of hypoxic exposure for promoting longevity while minimizing adverse effects remain to be determined [23]. Thus, careful consideration of the potential benefits and risks associated with hypoxia-induced longevity is crucial for developing the safe and effective therapeutic strategies targeting hypoxia signaling pathways in aging and age-related diseases.

Efforts to determine the optimal level of oxygen restriction for maximizing longevity and healthspan are underway, driven by the potential therapeutic benefits of hypoxia-induced adaptations. These involve assessing the effects of different hypoxic environments, ranging from mild to severe, on physiological function, oxidative stress levels, and disease susceptibility. By systematically manipulating oxygen levels and monitoring the resulting effects on cellular and organismal health, the future work should aim to identify the ideal balance between oxygen restriction and physiological adaptation for promoting longevity. Moreover, advances in technologies for measuring tissue oxygenation, such as hypoxia imaging techniques and oxygen-sensitive probes [299-302], enable researchers to precisely control and monitor oxygen levels *in vivo*, facilitating the identification of optimal oxygen conditions for maximizing healthspan and lifespan. By elucidating the ideal level of oxygen restriction for maximum benefit, the targeted interventions can be developed to promote healthy aging and improve overall well-being in aging populations.

Understanding the organ-specific effects of chronic continuous hypoxia is crucial for elucidating its impact on longevity and overall health. While hypoxia triggers adaptive responses that promote stress resistance and cellular survival, the extent and nature of these responses may vary across different organs and tissues [303-306]. Studies have shown that specific organs like the heart and brain are particularly vulnerable to hypoxic damage due to their high metabolic demands and oxygen sensitivity [297]. In contrast, other organs may exhibit greater resilience or even adaptive responses to chronic hypoxia. Investigating the organ-specific effects of chronic continuous hypoxia involves assessing changes in gene expression, metabolic activity, and tissue morphology to delineate the underlying mechanisms governing cellular adaptation and resilience. By elucidating the organ-specific effects of chronic hypoxia, research can provide insights into the complexities of hypoxia-induced longevity and tailor interventions to target specific organs or tissues for optimal healthspan extension.

## 8. Conclusions and future perspective

In summary, we reviewed the potential of hypoxia in promoting healthy aging and longevity. While the role of HIF in mediating cellular responses to hypoxia is well-established, our understanding of the broader molecular mechanisms governing hypoxia-induced longevity will continue to be investigated. The HIF-1 is a double-edged sword that can both promote and limit longevity via mechanistically distinct pathways [307]. HIF-1 plays a central role in the cellular response to low oxygen conditions, however, its impact on lifespan appears context-dependent. While promising, current evidence suggests a complex and nuanced picture. Activation of HIF-1 can be triggered by numerous extracellular stimuli, facilitating its nuclear translocation and subsequent transactivation of over 1000 genes [308-310]. To date, investigations into the lifespan-extending properties of HIF-1 have predominantly occurred in invertebrate models, such as the roundworm *C. elegans* and fruit fly. Although a singular study in mice (*Ercc1*^Δ/-^ mice) suggests that hypoxia may extend lifespan in the context of aging, the absence of discernible HIF activation signatures, along with negligible impacts on food intake, DNA damage markers, or senescence-related factors, indicates that hypoxia operates through unidentified downstream mechanisms [148].

While existing evidence highlights the potential benefits of hypoxia-induced longevity, further research is imperative to comprehensively elucidate its underlying mechanisms and optimize clinical application. Future inquiries should concentrate on delineating how the hypoxic response pathway enhances healthspan and lifespan in mammalian models, particularly emphasizing the distinct effects of HIF-1 on longevity versus disease progression. Given the evolutionary conservation of stress response pathways like HIF-1, it is plausible that some downstream effectors observed in invertebrates will also be present in mammals. Characterizing these downstream genes, along with identifying pertinent tissues and refining techniques for studying post-translational modifications, will be pivotal steps toward understanding HIF-1-mediated longevity and its translational potential. Moreover, efforts should focus on fully characterizing the HIF-1 signaling cascade and its downstream effectors, with a long-term goal of transitioning this knowledge into mammalian models. Such endeavors hold significant promise for delineating the role of stress response pathways in longevity and unraveling the intricate interplay between the HIF pathway and other longevity-associated factors. However, validating findings from hypoxia-induced longevity research across diverse aging models and human populations is essential for ensuring the generalizability and validity of conclusions. Furthermore, understanding the complex crosstalk between HIF-1 and other molecular pathways implicated in longevity underscores the multifaceted nature of longevity regulation. Finally, identifying optimal parameters for hypoxic exposure to maximize longevity benefits while minimizing adverse effects remains a critical area for exploration.

Despite distinct mechanistic pathways, both hypoxia and DR converge on common downstream effectors such as FOXO and FMOs to modulate lifespan. Yet, the context-dependent effects of each pathway on lifespan regulation underscore the need for nuanced investigation. The discovery of these downstream effectors holds promise for developing future interventions and therapeutics that not only extend lifespan but also mitigate the deleterious effects of hypoxia. Ultimately, insights gleaned from these endeavors may inform strategies for utilizing the HIF-1 pathway to enhance lifespan and healthspan in humans. In the coming years, as our understanding of the molecular mechanisms underlying hypoxia-induced longevity continues to evolve, it is likely that novel clinical opportunities for promoting healthy aging and extending lifespan will emerge. By leveraging insights gained from basic research into hypoxia-induced mechanisms, future therapeutic interventions may be developed to enhance both lifespan and healthspan in human populations. Thus, the exploration of hypoxia and its implications for longevity represents a compelling avenue for future scientific inquiry, with the potential to significantly impact human health and longevity.

Hypoxia can be deliberately induced under controlled conditions to elicit certain physiological benefits. This concept has garnered attention, particularly in the fields of exercise science, altitude training, and dietary interventions. Inducing hypoxia can lead to several positive outcomes, including cardiovascular fitness, increased mitochondrial efficiency, and enhanced metabolic adaptation [154, 311] such as fat oxidation and improved glucose regulation [312-315]. The application of hypoxia in daily life, although primarily utilized by athletes and those engaged in high-intensity interval training (HIIT) [315], is also relevant to the general population in certain forms. For instance, tools such as altitude masks or hypoxic chambers can simulate high-altitude conditions during exercise [311, 316], allowing individuals to integrate hypoxic training into their routines. The relationship between hypoxia, exercise, and diet is noteworthy, as hypoxic training can enhance physical performance and endurance. For example, using altitude masks during aerobic activities like running or cycling can lead to improved fitness outcomes by mimicking high-altitude conditions [311, 316]. While dietary interventions alone do not induce hypoxia, restrictive diets, such as ketogenic diets, may mimic certain aspects of hypoxia by promoting fat oxidation [317]. The combination of a restrictive diet with hypoxic training might amplify the metabolic benefits, although this approach should be pursued with caution.

Despite the potential benefits, the deliberate induction of hypoxia is not without risks. Prolonged or excessive exposure to hypoxic conditions can lead to adverse effects, including dizziness, fatigue, and in severe cases, conditions such as altitude sickness or hypoxia-induced injuries. Therefore, any attempt to induce hypoxia should be carefully monitored, preferably under the guidance of a healthcare or fitness professional to mitigate potential risks.
